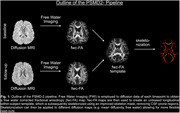# PSMD‐2: A fully automated marker for longitudinal assessment of cerebrovascular white matter injury

**DOI:** 10.1002/alz70856_100286

**Published:** 2025-12-25

**Authors:** Anna Dewenter, Benno Gesierich, Anna Kopczak, Saskia Lesnik Oberstein, Julie Rutten, Gido Gravesteijn, Nicolai Franzmeier, Hugh S Markus, Marco Duering

**Affiliations:** ^1^ Institute for Stroke and Dementia Research (ISD), University Hospital, LMU Munich, Munich, Bavaria, Germany; ^2^ Medical Image Analysis Center (MIAC) and Department of Biomedical Engineering, University of Basel, Basel, Switzerland; ^3^ Institute for Stroke and Dementia Research (ISD), University Hospital, LMU Munich, Munich, Germany; ^4^ LUMC, Leiden, Netherlands; ^5^ Institute for Stroke and Dementia Research (ISD), LMU University Hospital, LMU, Munich, Bavaria, Germany; ^6^ Munich Cluster for Systems Neurology (SyNergy), Munich, Bavaria, Germany; ^7^ Department of Psychiatry and Neurochemistry, University of Gothenburg, Mölndal, Västra Götalands län, Sweden; ^8^ University of Cambridge, Cambridge, United Kingdom

## Abstract

**Background:**

Cerebral small vessel disease (SVD) is highly prevalent in older adults and a key comorbidity in neurodegenerative conditions such as Alzheimer's disease. While conventional MRI markers (e.g. white matter hyperintensities) capture late SVD stages, diffusion MRI metrics are sensitive to early SVD‐related brain changes, making them key candidates as surrogate endpoints in clinical trials. The diffusion MRI marker “peak width of skeletonized mean diffusivity” (PSMD) has been used and validated in various SVD studies. Here, we developed this marker further and propose PSMD‐2, which is specifically tailored for longitudinal assessment. Improvements over the prior version include a) skeletonization using free‐water corrected diffusion maps, b) using a within‐subject template and a common skeleton across all timepoints, and c) allowing flexible use of multiple diffusion parameters (see Figure 1).

**Method:**

For PSMD‐2 development, we included *n* = 120 subjects with familial SVD (CADASIL from the DiViNAS study; 3T Philips, 2‐years follow‐up). For validation, we included *n* = 17 CADASIL patients (VASCAMY study; 3T Siemens, 18‐months follow‐up) and *n* = 72 patients with a history of lacunar stroke (“sporadic” SVD from the SCANS study; 1.5T GE, 2‐years follow‐up). PSMD‐2 was benchmarked against the conventional PSMD version via estimating required sample sizes for a hypothetical clinical trial using PSMD or PSMD‐2 as surrogate endpoints (power=80%, alpha=5%, mean change=30%).

**Result:**

PSMD‐2 closely tracked disease progression in all samples and yielded smallest sample size estimates in the CADASIL development dataset (24.3% reduction) as well as in both validation datasets (CADASIL: 31.0% reduction; sporadic SVD: 18.6% reduction). Validation in cerebral amyloid angiopathy (CAA) and instrumental validation addressing repeatability and reproducibility are ongoing.

**Conclusion:**

PSMD‐2 provides improved sensitivity to detect SVD‐related brain changes compared to the previous PSMD version, leading to smaller required sample sizes for clinical trials. Thus, PSMD‐2 is a key candidate as surrogate endpoint in SVD‐targeting trials, for monitoring disease progression and for capturing comorbid vascular brain changes in AD in longitudinal settings.